# The High Coercivity Field in Chemically Bonded WSe_2_/MoSe_2_ Powder

**DOI:** 10.3390/nano11123263

**Published:** 2021-12-01

**Authors:** Shiu-Ming Huang, Pin-Cyuan Chen, Pin-Cing Wang

**Affiliations:** 1 Department of Physics, National Sun Yat-sen University, Kaohsiung 80424, Taiwan; aa123412321@gmail.com (P.-C.C.); ryanw050616@gmail.com (P.-C.W.); 2 Center of Crystal Research, National Sun Yat-sen University, Kaohsiung 80424, Taiwan

**Keywords:** coercivity field, ferromagnetism, two-dimensional transition-metal dichalcogenide, WSe_2_, MoSe_2_

## Abstract

We studied the magnetic properties of WSe${}_{2}$/MoSe${}_{2}$ powder. The coercivity field reaches 2600 Oe at 5 K, 4233 Oe at 100 K and 1300 Oe at 300 K. These are the highest values reported for two-dimensional transition metal dichalcogenides. This study is different from the widely reported vacancy and zigzag structure-induced ferromagnetism studies. Importantly, a Raman peak red shift was observed, and that supports the chemical bonding at the interface between WSe${}_{2}$ and MoSe${}_{2}$. The large coercivity field originates from the chemical bonding-induced structural distortion at the interface between WSe${}_{2}$ and MoSe${}_{2}$.

## 1. Introduction

Spintronics is an approach to the manipulation of spin polarization and to realizing spin-base functionalities [1]. The dilute magnetic semiconductor (DMS) is one of the promising materials for spintronics applications. The original idea is doping a magnetic element into a semiconductor host, thereby making a material possessing both semiconductor and magnetic behaviors. The DMS has been widely studied in III–V and II–VI group semiconductor based systems, and has intrinsic ferromagnetism. However, the low Curie temperature and the intrinsic/extrinsic mechanism disputation limit its application potential. The combination of strong spin-orbit coupling and surface bonding has been shown to be very effective at generating magnetism in nanoparticles of metals [2] and semiconductors [3].

Two-dimensional transition-metal dichalcogenides (2D TMDs) have strong spin-orbit coupling and exhibit semiconductor behavior with an appropriate tunable bandgap [1,4,5,6]. Theoretical and experimental works have demonstrated that magnetism can be induced through doping magnetic elements, structure defects, or edge manipulation [7,8,9,10,11,12,13,14,15,16,17,18,19,20,21,22,23,24,25,26]. Reports show that the low coercivity field and oxidation in the MoS${}_{2}$ and WS${}_{2}$ with various element dopings or physical treatments would be too abrupt for applications. Differently from the broadly studied MoS${}_{2}$ and WS${}_{2}$, WSe${}_{2}$ and MoSe${}_{2}$ exhibit resistance to oxidation and humid ambiance [27,28]. WSe${}_{2}$ and MoSe${}_{2}$ also have a stronger spin-orbit interaction than MoS${}_{2}$ and WS${}_{2}$, and that might enhance the spin manipulation efficiency. However, there are rare reports about ferromagnetism of WSe${}_{2}$ and MoSe${}_{2}$. Both WSe${}_{2}$ and MoSe${}_{2}$ have the same crystallographic structure where 2D sheets are bounded in 3D stacks by van der Waals interactions. Experimental studies have reported room temperature ferromagnetism in Co, Ni and V-doped WSe${}_{2}$ [29,30,31,32,33,34]. However, the coercivity fields and ferromagnetism were still weak. A recent report revealed that the Co and Nb co-doped WSe${}_{2}$ has a strong coercivity field and magnetization, and it reached 1.2 kOe and 60.62 emu/g in the 1% Nb–4% Co co-doped WSe${}_{2}$ at 10 K [35]. It is understood that the vacancy and/or defect-induced pinning effect leads to the high coercivity and magnetization. Similarly to the magnetic element dopant, a theoretical calculation suggests that the ferromagnetism can be induced via structural defects (W or Se vacancies), and such structural defects could be achieved experimentally.

The structural defects and/or edge band bonding could lead to structural distortion that would induce ferromagnetism [36,37]. Recent studies have shown that element replacement might induce intrinsic ferromagnetism. It is interesting to know how it would be in mixed TMD materials. In this work, we demonstrate the thermally annealed WSe${}_{2}$/MoSe${}_{2}$ mixed powder. The WSe${}_{2}$ and MoSe${}_{2}$ blocks were chemically bonded. Our experimental results show high coercivity, 1324 Oe (2695 Oe) at room temperature (5 K). This is the highest coercivity ever reported in a TMD system.

## 2. Experimental Methods

The mixed WSe${}_{2}$/MoSe${}_{2}$ powder is a commercial product and was purchased from SixCarbon Technology. Co. (ShenZhen, China) The purchased WSe${}_{2}$/MoSe${}_{2}$ powder was vacuum-sealed in a glass tube with a pressure of 10${}^{-3}$ torr, and then thermally annealed. The WSe${}_{2}$/MoSe${}_{2}$ powder was heated up to 1000 ${}^{\circ }$C by a rate of 2.7 ${}^{\circ }$C/min, and maintained at 1000 ${}^{\circ }$C for 1 h. After thermal annealing, it was naturally cooled down to room temperature. The X-ray diffraction (XRD) was performed in D2 phaser using the Cu Kα radiation with a scan step of 0.1${}^{\circ }$. Raman spectroscopy was performed in the HORIBA, HR 800 (HORIBA Taiwan, Inc., Zhubei City, HsinChiu county, Taiwan) with wavelength 633 nm, power 16 mW and step 0.3 cm${}^{-1}$. X-ray photoelectron spectroscopy (XPS) was performed in ULVAC-PHI, PHI 5000 Versa Probe (ULVAC-PHI, Inc., Kanagawa ken, Japan), and used to detect the sample’s phase composition. The electron probe micro-analyzer (EPMA, JEOL Ltd., Musashino, Akishima, Tokyo, Japan) was performed in JEOL, JXA-8530F (JEOL, akishima Japan) and used to determine the material composition ratio. Magnetism measurements were performed using the standard technique in a SQUID MPMS-3 magnetometer (Quantum Design North America, Pacific Center Court, San Diego, CA, USA) in the temperature range of 5 to 300 K under an applied magnetic field of up to 5 T. The magnetic field step was 1000 Oe for sample 1 and 50 Oe for sample 2.

## 3. Results and Discussion

**Figure 1:**
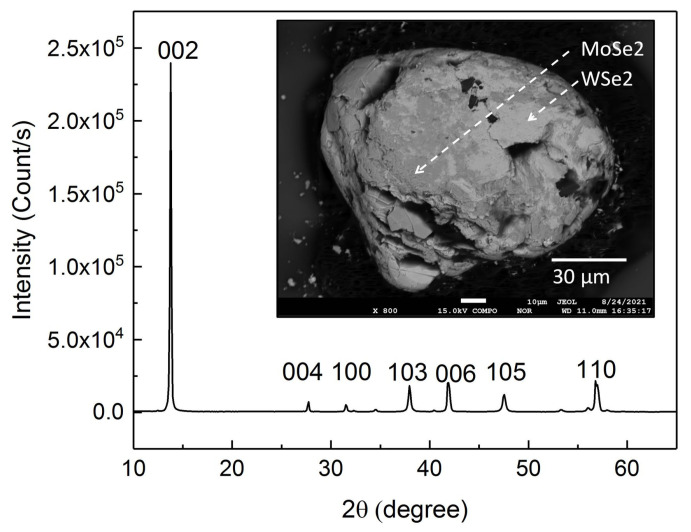
The XRD spectrum of the WSe${}_{2}$/MoSe${}_{2}$ powder. The peak position is consistent with the database. The sharp peaks imply that the sample is highly crystallized. The top-right inset shows that SEM image in the backscattering emission image mode. The light area is the WSe${}_{2}$, and the dark area is the MoSe${}_{2}$. The phases of WSe${}_{2}$ and MoSe${}_{2}$ are separated.

Figure 1 shows the XRD spectrum of the WSe${}_{2}$/MoSe${}_{2}$ powder. It shows sharp peaks and these peaks are consistent with the data on WSe${}_{2}$/MoSe${}_{2}$ powder [38]. The hexagonal structure is consistent with the structure of WSe${}_{2}$ and MoSe${}_{2}$. The XRD peak intensity over background noise reached 440 for the (002) peak, and the full-width at half-maximum (FWHM) was 0.2${}^{\circ }$. These results support that the WSe${}_{2}$/MoSe${}_{2}$ powders are highly crystallized. The crystallographic structure of WSe${}_{2}$ and MoSe${}_{2}$ domains might orient the same way in the whole grain. Figure 1 inset shows the SEM image of the WSe${}_{2}$/MoSe${}_{2}$ powder in backscattered emission imaging (BEI) mode. It reveals zones with different black and white intensity. The energy dispersive spectroscopy (EDS) supports that the light zone is WSe${}_{2}$ and the dark zone is MoSe${}_{2}$. It shows that there are no obvious cracks or geometric gaps between light zones and dark zones. Figure 1 inset exhibits that the WSe${}_{2}$ and MoSe${}_{2}$ are mainly individual blocks and do not appear in the WSe${}_{2-x}$Te${}_{x}$ form, which would lead to a wide range of gray intensity in the BEI mode. The EPMA supports that W:Se = 1:2 in the light zone and Mo:Se = 1:2 in the dark zone, and ${\mathrm{MoSe}}_{2}$:${\mathrm{WSe}}_{2}\approx$ 1:1.

**Figure 2:**
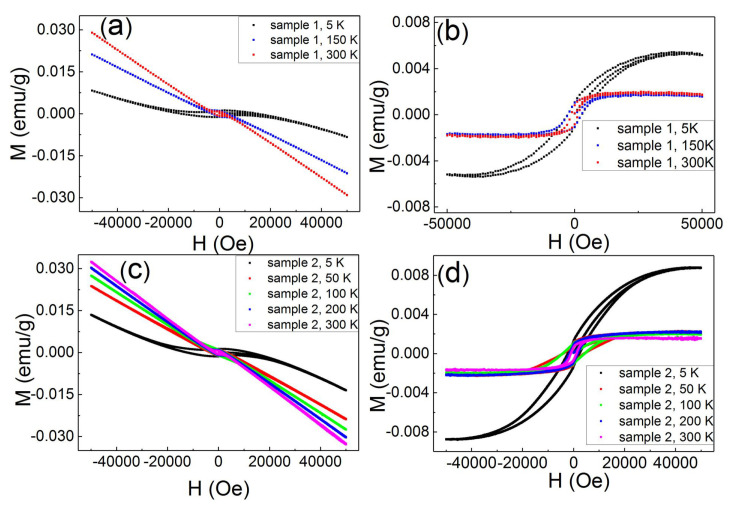
(**a**,**c**) The M–H curves at temperatures for sample 1 and sample 2. They exhibit hysteresis loops at low magnetic fields and diamagnetism at high magnetic fields. (**b**,**d**) The M–H curves at temperature for sample 1 and sample 2; the diamagnetic contribution was subtracted.

Figure 2a shows the WSe${}_{2}$/MoSe${}_{2}$ magnetization as a function of magnetic fields and it reveals the diamagnetization at high magnetic fields. The M–H loop shows a clear hysteresis loop, and that is a ferromagnetism feature. The diamagnetic background is superimposed onto the ferromagnetic loop. The ferromagnetism is known to be saturated at critical magnetic fields, and the diamagnetism is negatively linearly correlated with magnetic fields. After subtracting out the diamagnetic contribution that is determined from the magnetism at high magnetic fields, one could extract the ferromagnetism signal. Figure 2b shows the extracted ferromagnetic loops. The coercivity field was 1300 Oe at 300 K and 2600 Oe at 5 K, and these coercivity fields are larger than all values reported for 2D TMDs. To further confirm this large coercivity field, the other sample was prepared from the same raw material and under the same treatment conditions. Figure 2c shows that the M–H loop shows ferromagnetic loops for the second sample. The M–H curve is similar to the curve of the first sample. Figure 2d exhibits ferromagnetic loops after subtracting out the diamagnetic background. It exhibits the ferromagnetic features with a coercivity field of 1100 Oe at 300 K and 2299 Oe at 5 K. This is consistent with the results of the first sample, and confirms that this large coercivity is an intrinsic feature.

**Figure 3:**
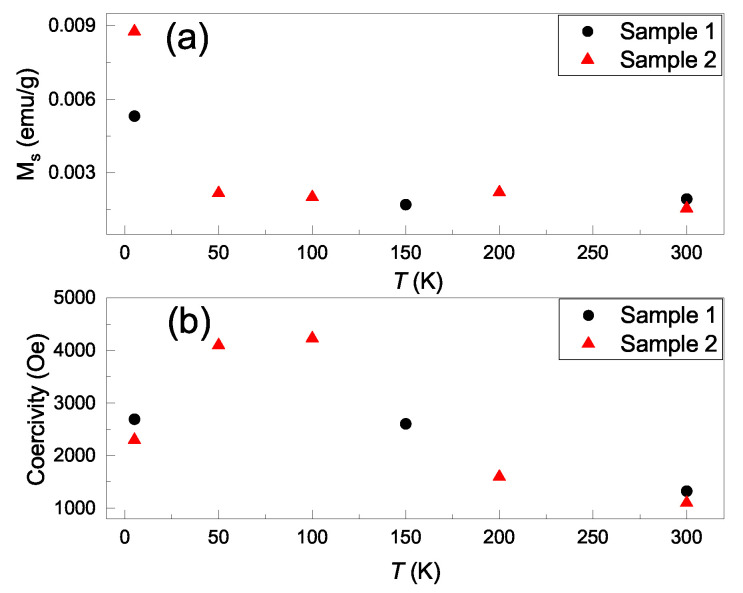
(**a**) The saturation magnetization as a function of temperature for two WSe${}_{2}$/MoSe${}_{2}$ powders. (**b**) The temperature dependent coercivity fields of two WSe${}_{2}$/MoSe${}_{2}$ powders.

Figure 3a shows the saturation magnetization as a function of temperature, and it reveals consistent values in the two samples. The saturation magnetization is roughly consistent with the values reported for 2D TMDs. Figure 3b shows the temperature-dependent coercivity fields. It shows a smooth curve and a maximum value of 4233 Oe at 100 K. Table 1 lists the reported saturation magnetization and coercivity fields in 2D materials. These hysteresis loop coercivity fields have a wide range of values. Our observations of 2600 Oe at 5 K, 4233 Oe at 100 K and 1300 Oe at 300 K are the largest values reported for 2D TMDs at those temperatures.

A slight magnetic or transition element dopant might lead to strong ferromagnetism in 2D TMDs [29,30,31,32,33,34]. Our EDS analysis supports that there were no un-avoided magnetic or transition elements in our system. The saturation magnetization was 0.001 emu/g. If this magnetism originated from Ni, Co or/and Fe, the magnetic elements would have reached a 0.01% atomic ratio, which is within the detectable range of the EMPA, but our EMPA experiment showed no detectable magnetic elements in our samples. This supports that the external element dopants are not the main mechanism of the observed ferromagnetism in WSe${}_{2}$/MoSe${}_{2}$ powder.

Apart from the magnetic element dopants, theoretical calculations and experimental work support that the structural defects can induce ferromagnetism. The coercivity field is sensitive to the host material, number of defects and defect structure [7,8,9,10,11,12,13,14,15,16,17,18,19,20,21,22,23,24,25,26]. The vacancies and defects were expected to be uniformly distributed in the entirety of WSe${}_{2}$-MoSe${}_{2}$ blocks, and not only in specific one material (WSe${}_{2}$ or MoSe${}_{2}$). Table 1 shows that the defect-induced coercivity field in WSe${}_{2}$ was roughly one order of magnitude higher than that in MoSe${}_{2}$. In a case where the observed hysteresis loop originates from the vacancy or defect in the WSe${}_{2}$ and MoSe${}_{2}$, one would expect to observe two hysteresis loop steps in our samples. Only one hysteresis loop was observed though; see Figure 2. On the other hand, we report that the thermal annealing induced S vacancies in WS${}_{2}$ and MoS${}_{2}$. The XPS shows no obvious Mo, W and Se vacancies in the WSe${}_{2}$/MoSe${}_{2}$ powder. The structure of vacancies might impact the XRD peak intensity suppression and XRD peak shift. As mentioned in Figure 1, the XRD peaks are extremely sharp and show no XRD peak shift. These results indicate extremely little vacancy in our system, and we do not think that this slight, unavoidable structural defect could have led to the large coercivity fields observed. This implies that the structural defects are not the dominant mechanism.

Ferromagnetism was studied in the MoS${}_{2-x}$Se${}_{x}$ nanosheet, and the results revealed that the ferromagnetism is sensitive to the Se/S ratio. MoS${}_{2-x}$Se${}_{x}$ exhibited the most ferromagnetism in the Mo(S${}_{0.49}$Se${}_{0.51}$)${}_{2}$ nanosheet, in which the Se/S ratio was the largest [36]. The magnetism decreased as more Se or S were substituted into the Mo(S${}_{0.49}$Se${}_{0.51}$)${}_{2}$ nanosheet. This supports the idea that the element replacement might lead to the ferromagnetism. The chemical bonding at the WSe${}_{2}$ and MoSe${}_{2}$ interface would be in the WSe${}_{2-x}$Te${}_{x}$ form. This would lead to structural distortion, and the observed hysteresis loop might originate from the interface. On the other hand, it is reported that ferromagnetism and magnetoresistance hysteresis can be observed in a molecular-beam epitaxy grown non-magnetic group IV Ge${}_{1-x}$Sn${}_{x}$ thin film. A Ge${}_{1-x}$Sn${}_{x}$ alloy forms at the interface between Ge and Sn thin films. The observed ferromagnetism is understood as the inversion symmetry breaking from atomic disordering in the alloy [37].

**Figure 4:**
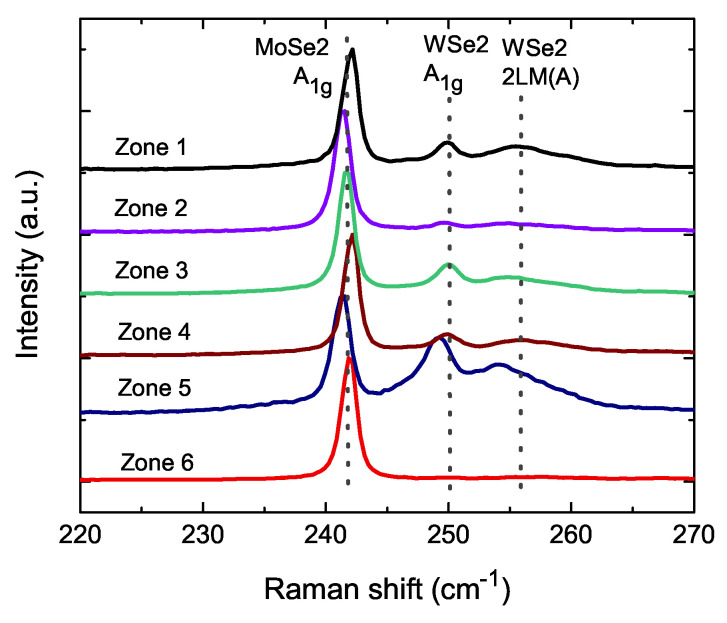
The Raman shifts of two WSe${}_{2}$/MoSe${}_{2}$ powders in different zones. We exhibit the standard peaks of WSe${}_{2}$ and MoSe${}_{2}$. Red shifts of WSe${}_{2}$ peaks are shown in zone 5.

The Raman spectrometer, a sensitive tool for detecting lattice bonding, was used to identify the chemical bonding at the interface between WSe${}_{2}$ and MoSe${}_{2}$. Figure 4 shows the Raman spectra of different zones. The WSe${}_{2}$/MoSe${}_{2}$ powder size was two orders of magnitude larger than the Raman laser spot dot size of about 1 μm. The Raman spectra might have detected the signal of only WSe${}_{2}$, MoSe${}_{2}$ or chemically bonded WSe${}_{2}$/MoSe${}_{2}$. That means the Raman spectra exhibit slight different peak positions and peak intensity at different zones. The spectra are consistent with the database of WSe${}_{2}$ and MoSe${}_{2}$. The peaks of MoSe${}_{2}$ A${}_{1g}$ (242 cm${}^{-1}$), WSe${}_{2}$ A${}_{1g}$ (250 cm${}^{-1}$) and WSe${}_{2}$ 2LA(M) (256 cm${}^{-1}$) were labeled with dotted lines. It is noticeable that there are different peaks positions of WSe${}_{2}$ in zone 5. The peak positions are 253 and 249 cm${}^{-1}$ in zone 5. The measured step was 0.3 cm${}^{-1}$, which is much smaller than the peak difference in the A${}_{1g}$ and 2LA(M), and the red shifting of these peaks might have originated from the intrinsic lattice vibration mode in the WSe${}_{2}$/MoSe${}_{2}$ powder. It also shows a larger red shift in the W${}_{1-x}$Mo${}_{x}$Se${}_{2}$ with more Mo replacement [39]. As shown in the Figure 1 inset, there is a merge combination. The peaks at 253 and 249 cm${}^{-1}$ were expected to have red shifts: peak 2LA(M) to 256 cm${}^{-1}$ for A${}_{1g}$, and the 250 cm${}^{-1}$ peak for WSe${}_{2}$. This is evidence of the chemical bonding in the WSe${}_{2}$/MoSe${}_{2}$ powder. Focusing on the MoS${}_{2-x}$Se${}_{x}$ nanosheet, our WSe${}_{2}$/MoSe${}_{2}$ powder is a three-dimensional chemical bonding system. Compared to the nanosheet, a three-dimensional system would possess more interface chemical bonding between WSe${}_{2}$ and MoSe${}_{2}$, and that would greatly enhance the total amount of structural distortion. This could have led to large coercivity fields in our WSe${}_{2}$/MoSe${}_{2}$ powder.

**Figure 5:**
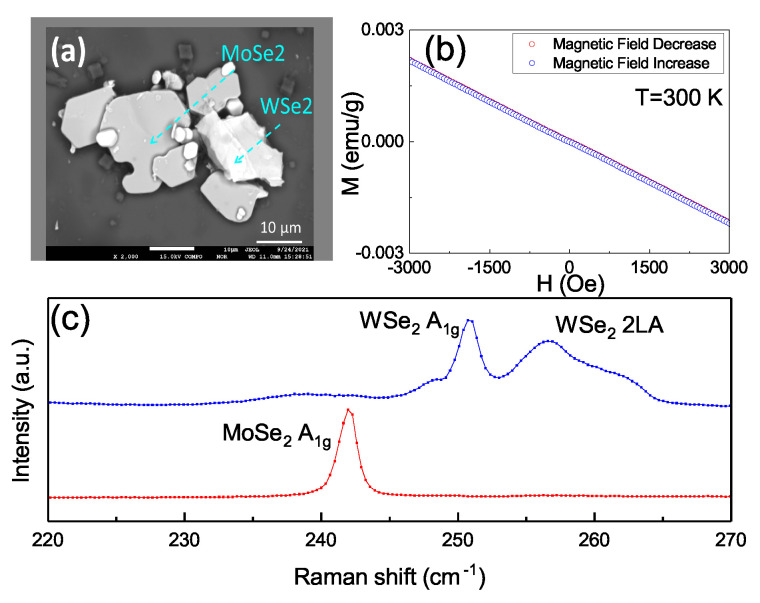
(**a**) The SEM image in the backscattering emission imaging mode. The light area is the WSe${}_{2}$ and the dark area is the MoSe${}_{2}$. The sample was a mixture of WSe${}_{2}$ and MoSe${}_{2}$ flakes containing only one material within each flake. (**b**) The M–H curve shows the diamagnetism feature, and no hysteresis loops were detected. (**c**) The 242 cm${}^{-1}$ for MoSe${}_{2}$, and 250 cm${}^{-1}$ and 256 cm${}^{-1}$ for WSe${}_{2}$, are shown in the Raman spectra. No mixed peaks or red-shifted peaks were observed.

To further identify the source of the observed ferromagnetism, another mixed WSe${}_{2}$ and MoSe${}_{2}$ powder from the same raw materials was prepared. The sample was a mixture of WSe${}_{2}$ and MoSe${}_{2}$ flakes containing only one material within each flake. Figure 5a shows these MoSe${}_{2}$ and MoSe${}_{2}$ were individually distributed with no geometric connection between two materials. Figure 5b shows a diamagnetic feature with the backward and forward magnetic field strength from −3000–0 Oe and 0–3000 Oe, respectively. The backward and forward sweeping completely overlap and no hysteresis loops were detected—indicating the absence of ferromagnetism in the sample. Figure 5c shows that only individual peaks of 242 cm${}^{-1}$ for MoSe${}_{2}$, and 250 cm${}^{-1}$ and 256 cm${}^{-1}$ for WSe${}_{2}$ were observed in the Raman spectra. No mixed peaks or red-shifted peaks were observed. This shows that the individual MoSe${}_{2}$ or WSe${}_{2}$ in our source material would not lead to the ferromagnetism. This supports the sample having no ferromagnetism due to the lack of chemical bonding between WSe${}_{2}$ and MoSe${}_{2}$ blocks, so the ferromagnetism might originate from the structural distortion at the interface between WSe${}_{2}$ and MoSe${}_{2}$.

## 4. Conclusions

An investigation of the magnetism of WSe${}_{2}$/MoSe${}_{2}$ powder was performed. The coercivity field reaches 2600 Oe at 5 K, 4233 Oe at 100 K and 1300 Oe at 300 K. These are the largest values reported for two-dimensional transition metal dichalcogenides, distinguishing them from the widely reported vacancy and zigzag structure-induced ferromagnetism values. A Raman peak red shift was observed, which supports the chemical bonding at the interface of WSe${}_{2}$ and MoSe${}_{2}$. The large coercivity field originates from the chemical bonding-induced structural distortion at the interface between WSe${}_{2}$ and MoSe${}_{2}$.

## Figures and Tables

**Table 1:** List of the reported coercivity and saturation magnetization values of two-dimensional transition metal dichalcogenides.

Material	Coercivity	Saturation Magnetization	Temperature	Treatment	Reference
WSe${}_{2}$/MoSe${}_{2}$ powder	2695 Oe	0.0053 emu/g	5 K	interface	This work
WSe${}_{2}$/MoSe${}_{2}$ powder	2606 Oe	0.0017 emu/g	150 K	interface	This work
WSe${}_{2}$/MoSe${}_{2}$ powder	1324 Oe	0.0019 emu/g	300 K	interface	This work
WSe${}_{2}$/MoSe${}_{2}$ powder	2299 Oe	0.0087 emu/g	5 K	interface	This work
WSe${}_{2}$/MoSe${}_{2}$ powder	4100 Oe	0.0022 emu/g	50 K	interface	This work
WSe${}_{2}$/MoSe${}_{2}$ powder	4233 Oe	0.0020 emu/g	100 K	interface	This work
WSe${}_{2}$/MoSe${}_{2}$ powder	1600 Oe	0.0022 emu/g	200 K	interface	This work
WSe${}_{2}$/MoSe${}_{2}$ powder	1100 Oe	0.0015 emu/g	300 K	interface	This work
WSe${}_{2}$ nanosheet	414 Oe	211 emu/cm${}^{3}$	20 K	edge	Ref. [7]
WSe${}_{2}$ naosheet	106 Oe	70 emu/cm${}^{3}$	300 K	edge	Ref. [7]
WSe${}_{2}$ few-layer	578 Oe	0.078 emu/g	10 K	zigzag	Ref. [8]
WSe${}_{2}$ few-layer	200 Oe	0.0073 emu/g	300 K	zigzag	Ref. [8]
WS${}_{2}$ nanosheet	1115 Oe	0.0046 emu/g	3 K	zigzag	Ref. [9]
WS${}_{2}$ nanosheet	92 Oe	0.0052 emu/g	300 K	zigzag	Ref. [9]
WS${}_{2}$ nanosheet	240 Oe	0.39 emu/g	10 K	zigzag and structure defect	Ref. [10]
WS${}_{2}$ nanosheet	140 Oe	0.2 emu/g	300 K	zigzag and structure defect	Ref. [10]
WS${}_{2}$ exfoiled nanosheet	400 Oe	0.004 emu/g	10 K	zigzag or vacancy	Ref. [11]
WS${}_{2}$ exfoiled nanosheet	125 Oe	0.002 emu/g	300 K	zigzag or vacancy	Ref. [11]
WS${}_{2}$ few-layer	295 Oe	0.098 emu/g	10 K	zigzag	Ref. [8]
WS${}_{2}$ few-layer	130 Oe	0.009 emu/g	300 K	zigzag	Ref. [8]
WS${}_{2}$ powder	∼60 Oe	0.002 emu/g	300 K	vacancy	Ref. [12]
WS${}_{2}$ nanoflake	293 Oe	3.67 emu/g	60 K		Ref. [13]
WS${}_{2}$ nanoflake	171 Oe	1.82 emu/g	300 K		Ref. [13]
WS${}_{2}$ nanoflake	967 Oe	7.59 emu/g	60 K		Ref. [13]
WS${}_{2}$ nanoflake	239 Oe	3.08 emu/g	300 K		Ref. [13]
MoSe${}_{2}$ nanoflake	100 Oe	1.39 emu/g	300 K	zigzag	Ref. [14]
MoSe${}_{2}$ few-layer	435 Oe	0.013 emu/g	10 K	zigzag	Ref. [8]
MoSe${}_{2}$ few-layer	40 Oe	0.0026 emu/g	300 K	zigzag	Ref. [8]
MoSe${}_{2}$ nanoflowers	50 Oe	0.027 emu/g	300 K	thermal vacancy	Ref. [15]
MoSe${}_{2}$ nanoflowers	80 Oe	0.017 emu/g	300 K	thermal vacancy	Ref. [15]
MoSe${}_{2}$ nanoflowers	60 Oe	0.003 emu/g	300 K	thermal vacancy	Ref. [15]
MoS${}_{2}$ nanosheet	150 Oe	1 emu/g	300 K	vacancy	Ref. [16]
MoS${}_{2}$ 1T phase	150 Oe	12.5 emu/g	300 K	structure phase	Ref. [16]
MoS${}_{2}$ nanosheets	50∼200 Oe	0.1 emu/g	5 K	thermal vacancy	Ref. [17]
MoS${}_{2}$ nanosheets	20∼50 Oe	0.008 emu/g	300 K	thermal vacancy	Ref. [17]
MoS${}_{2}$ film	260 Oe	0.00125 emu/cm${}^{3}$	300 K	proton irradiation	Ref. [18]
MoS${}_{2}$ film	700 Oe	0.0015 emu/cm${}^{3}$	10 K	proton irradiation	Ref. [18]
MoS${}_{2}$ film	276 Oe	0.0486 emu/g	300 K	web buckle-mediated strain	Ref. [19]
MoS${}_{2}$ nanoparticles	20.8 Oe	0.1 emu/g	5 K	thermal vacancy	Ref. [20]
MoS${}_{2}$ nanosheet	241.3 Oe	1.08 emu/g	10 K	zigzag and structure vacancy	Ref. [10]
MoS${}_{2}$ nanosheet	∼80 Oe	0.8 emu/g	300 K	zigzag and structure vacancy	Ref. [10]
MoS${}_{2}$ single crystal bulk	400 Oe	0.004 emu/g	50 K	zigzag	Ref. [21]
MoS${}_{2}$ single crystal bulk	100 Oe	0.0038 emu/g	300 K	zigzag	Ref. [21]
MoS${}_{2}$ pyramid (films)	∼200 Oe	3 emu/g	2 K	zigzag	Ref. [22]
MoS${}_{2}$ pyramid (films)	∼50 Oe	2.9 emu/g	300 K	zigzag	Ref. [22]
MoS${}_{2}$ nanosheet	∼55 Oe	0.01 emu/g	300 K	S vacancy and substitutional dopants	Ref. [23]
MoS${}_{2}$ 1T phase	200 Oe	0.057 emu/g	5 K	electron beam formed defects	Ref. [24]
MoS${}_{2}$ nanosheet	∼200 Oe	0.0073 emu/g	300 K	un-paired Mo or edge	Ref. [25]
MoS${}_{2}$ few-layer	517 Oe	0.019 emu/g	10 K	zigzag	Ref. [8]
MoS${}_{2}$ few-layer	146 Oe	0.0043 emu/g	300 K	zigzag	Ref. [8]
MoS${}_{2}$ nanoribbons	∼250 Oe	0.032 emu/g	2 K	zigzag	Ref. [26]
MoS${}_{2}$ nanoribbons	∼250 Oe	0.026 emu/g	300 K	zigzag	Ref. [26]
Co doped WSe${}_{2}$	515 Oe	6.89 emu/g	5 K	dopant	Ref. [20]
Co doped WSe${}_{2}$	400 Oe	5 emu/g	300 K	dopant	Ref. [20]
Ni doped WSe${}_{2}$	40 Oe	0.0067 emu/g	300 K	dopant	Ref. [29]
Co doped WSe${}_{2}$	465 Oe		3 K	dopant	Ref. [10]
Nb and Co codoped WSe${}_{2}$	1200 Oe	250 emu/cm${}^{3}$	10 K	dopant	Ref. [35]
Nb and Co codoped WSe${}_{2}$	0 Oe	150 emu/cm${}^{3}$	300 K	dopant	Ref. [35]
Co doped MoS${}_{2}$	400 Oe	0.025 emu/g	300 K	dopant	Ref. [30]
Ni doped MoS${}_{2}$	175 Oe	0.14 emu/g	300 K	dopant	Ref. [30]
Mn doped MoS${}_{2}$	1076 Oe	0.015 emu/g	50 K	dopant	Ref. [31]
V doped MoS${}_{2}$	1870 Oe	0.067 emu/g	10 K	dopant	Ref. [32]
V doped MoS${}_{2}$	81 Oe		300 K	dopant	Ref. [32]
N doped ReS${}_{2}$	1200 Oe	1.2 emu/g	20 K	dopant	Ref. [33]
Fe doped SnS${}_{2}$	400 Oe	3.5 emu/g	2 K	dopant	Ref. [34]

## Data Availability

The data presented in this study are available on request from the corresponding author.
